# Real-time, simultaneous myoelectric control using a convolutional neural network

**DOI:** 10.1371/journal.pone.0203835

**Published:** 2018-09-13

**Authors:** Ali Ameri, Mohammad Ali Akhaee, Erik Scheme, Kevin Englehart

**Affiliations:** 1 Department of Biomedical Engineering, Shahid Beheshti University of Medical Sciences, Tehran, Iran; 2 School of Electrical and Computer Engineering, University of Tehran, Tehran, Iran; 3 Institute of Biomedical Engineering, University of New Brunswick, Fredericton, NB, Canada; Queen Mary University of London, UNITED KINGDOM

## Abstract

The evolution of deep learning techniques has been transformative as they have allowed complex mappings to be trained between control inputs and outputs without the need for feature engineering. In this work, a myoelectric control system based on convolutional neural networks (CNN) is proposed as a possible alternative to traditional approaches that rely on specifically designed features. This CNN-based system is validated using a real-time Fitts’ law style target acquisition test requiring single and combined wrist motions. The performance of the proposed system is then compared to that of a standard support vector machine (SVM) based myoelectric system using a set of time-domain features. Despite the prevalence and demonstrated performance of these well-known features, no significant difference (*p*>0.05) was found between the two methods for any of the computed control metrics. This demonstrates the potential for automated learning approaches to extract complex and rich information from stochastic biological signals. This first evaluation of the usability of a CNN in a real-time myoelectric control environment provides a basis for further exploration.

## Introduction

Pattern recognition based myoelectric control has shown great potential in powered prostheses [1], and commercial implementations have become available in the past few years [2]. EMG pattern recognition can be broadly divided into classification ([3–5]) and regression ([6–12]) based methods, where either a classifier or regressor is trained to estimate the movement intent from multi-channel EMG signals [1, 13].

Because of the stochastic nature of EMG signals, instantaneous EMG values cannot be used to estimate motor intent [13]. Instead, a set of EMG features must be extracted from a time window (typically between 100-200ms [14, 15]), to be used for motor intent estimation. Many EMG features have been proposed in the literature of the decades. One of the most popular feature sets is known as the time domain set (TD) [16] and includes the mean absolute value (MAV), zero crossings, waveform length, and slope sign changes. The TD feature set contains information on both temporal and frequency content of EMG signal. Other popular EMG features in the literature include the mean frequency, auto-regressive coefficients, Willison amplitude, mean power, and histogram, among others. A review of EMG signal feature selection is provided in [17].

The proper design and selection of features can greatly impact the performance of motor intent estimation and has been a subject of much research [18]. Many of the EMG features proposed in the literature, are highly correlated, leading to feature redundancy [19]. Thus, it is important to obtain non-redundant features allowing maximum class separability, robustness and minimum complexity. Depending on the application, these features should be generalizable across various datasets [19].

Given their importance, it would be desirable to have a system capable of automatically identifying and extracting relevant features from the EMG signal. This ideal can be achieved by using deep learning methods such as deep neural networks, deep belief networks, and recurrent neural networks [20]. Recently, deep learning has revolutionized several fields such as computer vision, speech recognition, and bioinformatics, where they have produced results similar or superior to human experts [20].

**Fig 1:**
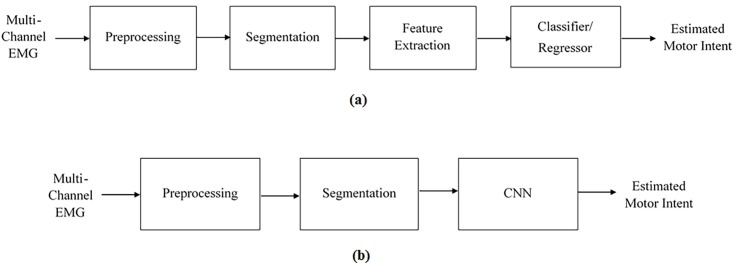
A simple block diagram of (a) classical and (b) CNN based approaches to myoelectric pattern recognition.

Convolutional neural networks (CNNs) are an example of a deep learning framework. Like other deep learning methods, CNNs are attractive as they can learn classification tasks directly from the raw data, eliminating the need for feature engineering and extraction [21]. CNNs are most useful in tasks where design and calculation of features are difficult, such as in facial recognition systems. The recent surge in the application of CNNs is also partially due to advances in GPU technology that has substantially reduced the model training time [20]. Fig 1 shows high-level block diagrams of classical and CNN based approaches to EMG pattern recognition.

A handful of recent studies have used CNNs to extract EMG patterns. Park *et al*. [22] and Atzori *et al*. [23] used CNNs to estimate hand movements directly from EMG signals. Geng *et al*. [24] applied CNNs to high density EMG data to classify hand gestures, using spatial EMG data matrices as the model input. Xia *et al*. [25] used Recurrent CNNs in the EMG frequency transform to classify hand movements. Allard *et al*. [26] used CNNs on the EMG spectrogram to classify hand/wrist gestures and to control a robotic arm to pick up a cube and place it in a specified location.

Other than in [26], however, previous work on CNN based EMG pattern recognition has been largely limited to offline studies. Because it is not clear how offline classification accuracy translates to controllability of a prosthesis, performance should be assessed using closed-loop (with visual feedback) real-time tests [27]. In this study, the use of CNNs is investigated for EMG based motor intent estimation. To determine the usability of the proposed method, the system control performance is assessed in a real-time test and is directly compared to that of a classical pattern recognition approach. The test consisted of a Fitts’ law [28] style virtual target acquisition task, which has been used previously for evaluation of myoelectric control [29–31, 9].

In an attempt to provide more natural and intuitive control, many recent works have studied simultaneous myoelectric control of multiple movements using classification [32–40] and regression [35–40] based methods. In this work, a CNN-based scheme for simultaneous myoelectric control of wrist movements is developed and compared to a classical pattern recognition system with engineered features in real-time target acquisition task.

## Methods

Seventeen able-bodied individuals (ages: 29.5±3.7 yrs, 15 right-handed, 2 left-handed) participated in this study in Feb, Mar 2018. Initially 20 subjects were approached, but 3 of them dropped out before or after the practice session. This study was approved by the institutional ethics board of Shahid Beheshti Medical University. Before the experiments, all subjects provided written informed consent. The subjects performed sufficient practice sessions on a different day, prior to the main session, to become familiar with the experiment. Eight pairs of bipolar surface electrodes (g.HiAmp, g-tec Inc.) were attached, equally spaced, around the dominant forearm proximal to the elbow. A reference electrode was placed on the ulnar styloid of the opposite wrist. A sampling rate of 1.2 KHz was used to record the EMG signals, which were then bandpass filtered between 5–500 Hz and notch filtered at 50 Hz using eighth-order Butterworth filters. Data acquisition and processing were performed using Matlab R2017b.

Eight wrist movements were investigated including flexion, extension, pronation, supination, and their simultaneous combinations: extension and pronation, extension and supination, flexion and pronation, and flexion and supination. The experiment comprised an initial training session followed by a set of real-time Fitts’ law tests.

### Training protocol

The training session consisted of nine trials, including a *no motion* trial, as well as eight dynamic trials corresponding to each of the wrist movements listed above. The *no motion* trial lasted 30s, while the users maintained a relaxed posture. During the other eight trials, the subjects were prompted to perform the trial-specific contraction dynamically with an intensity proportional to a progress bar on a computer screen [41, 42]. The dynamic contractions involved four repetitions of the following cycle: 3s of *no motion*, 3s of ramping up to the full-range contraction, 3s of maintaining the full-range contraction, followed by 3s of returning to *no motion*. For the full-range contraction, the users were asked to perform the corresponding contraction with a subjective comfortable medium intensity. Dynamic motions were used during training as they have been reported to improve classifier robustness [43].

### Machine learning

The EMG data recorded in the training session were processed offline as follows. Data from the active trials (2–9) were removed when the target was lower than 30% (determined empirically) of the full-range to avoid inadvertently including *no motion* data. The remaining EMG data were included as the corresponding wrist contraction. Each training trial was included as a separate class, to produce nine total classes. The EMG data were segmented using 160ms windows with an increment of 40ms.

Two different methods were employed for EMG-based motor intent estimation: A CNN based approach and a support vector machine (SVM) based approach, previously shown to perform well in online pattern recognition-based myoelectric control [44, 9].

For the CNN, the EMG data were first arranged to form 8x192 matrices, where eight is the number of channels and 192 is the number of samples in each window (160ms). The EMG matrices and their corresponding motion labels were used to train the CNN. Ninety percent of the data were selected randomly as the training set and the remaining data were used for validation.

**Fig 2:**
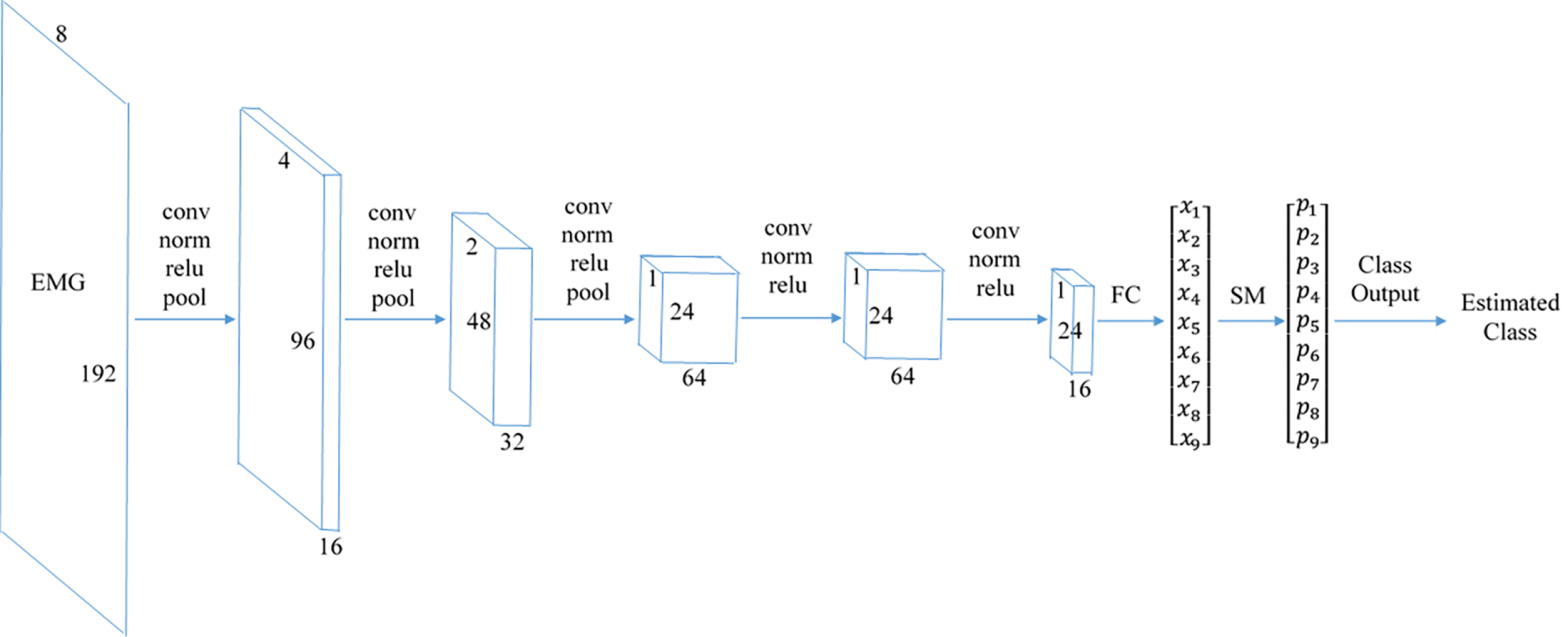
The extracted feature maps are shown during the proposed CNN process. The input is 8x192 EMG data. In each phase, the features are obtained by applying convolution, batch normalization, rectified linear unit (relu), and pooling. A fully connected (FC) layer combines the features to produce 9 final features. The softmax (SM) layer computes the classification probabilities, and the classification layer outputs the estimated class. (Note that for example 32 3x3x16 convolutions are performed in the second convolution layer, and the second pooling layer transforms the 4x96 feature map to 2x48 feature map).

The proposed CNN consisted of 22 layers including an input layer, five convolution layers, five batch normalization layers, five rectified linear unit (Relu) layers, three max-pooling layers, a fully connected layer, a softmax layer and a classification layer, illustrated in Fig 2. The basic architecture of the proposed CNN is similar to that of the well-known networks used in computer vision such as *AlexNet* [45, 46]. The exact configuration, however was determined empirically based on pilot studies with EMG acquired as described in the Methods section. The concepts of CNNs layers are detailed in [20].

Before each convolution, the data matrix was zero-padded. The number of filters in convolution layers 1 to 5 were 16, 32, 64, 64, and 16, respectively, and all filters were 3x3. Max-pooling was performed on 2x2 regions with a stride of two. A *Stochastic Gradient Descent with Momentum* (*SGDM*) algorithm with an initial learning rate of 0.001, was used for the network training. The network was validated twice per epoch.

The minibatch size was set to 128. Based on the pilot studies, it was found that decreasing the minibatch size lower than 128 did not improve the classification accuracy, but increased the training time. Also, increasing the minibatch size higher than 128, reduced the classification performance.

The number of epochs was set to 40. Based on the pilot studies, it was found that increasing the number of epochs higher than 40, did not improve the classification accuracy, but increased the training time. Also, decreasing the number of epochs lower than 40, reduced the classification performance.

To avoid CNN overfitting two procedures were performed:

- *L2* Regularization: A regularization term for the weights to the loss function was used to decrease overfitting. The regularization factor was set to 10^−4^ (found empirically).
- Early Stopping using validation set: If the error on the validation set increased for five iterations, the training was stopped. The network was validated twice per epoch.

It must be noted that the CNNs were not pretrained. For each subject, a CNN was trained from scratch. Only the configuration (such as number and type of layers) were determined in pilot studies.

The training was performed using an Nividia GTX 1050 graphics processing unit (GPU), and took on average 100s for each subject.

For the SVM-based system, five features, including the widely used TD set [16] as well as mean frequency, were calculated for each window. The TD set was employed as it is the most popular feature set in the literature. Also, pilot work showed that inclusion of the mean frequency improves the results.

The EMG features and their corresponding motion labels were used to train the 9-class SVM classifier. The SVM computation was performed using the *libsvm* package in Matlab [47]. The radial basis function kernel, $\gamma ={number\;of\;features}^{-1}=\frac{1}{40}$, and a cost parameter of 1 were selected empirically as they gave the best performance in pilot work.

### Fitts’ law test

A two-dimensional Fitts’ law style real-time target acquisition test [29–31] was conducted to assess the control performance of the CNN and SVM based systems. During the test, a fixed target was presented on a computer screen, and users were prompted to control a cursor to reach the target as quickly as possible. To acquire a target, the users had to match the horizontal position and orientation of the cursor with those of the target. *Velocity control* was employed so that the classifier output determined the cursor movement direction, and the user effort intensity was mapped to the cursor speed. For right-handed users, wrist extension and flexion moved the cursor to right and left, respectively, while wrist pronation and supination rotated the cursor orientation counterclockwise and clockwise, respectively. For left-handed users, these directions were reversed. Class outputs consistent with the combined motions moved the cursor position and rotated its orientation, simultaneously, in the corresponding direction.

As classifiers do not inherently accommodate proportional control, the following equation was employed to calculate the magnitude of the cursor speed, *V*, as a measure of user effort.

$$V=G∙\frac{X/Y-TH}{1-TH}$$ (1)

where *X* is the current MAV of the EMG averaged over all channels, and *Y* is the maximum of the same parameter over all windows in the current class of the training set. The threshold (*TH*) was used to prevent unwanted small movements of the cursor and was set to 0.2 (found empirically). This approach has been shown to facilitate control near zero speeds, despite the inclusion of a contraction intensity activation threshold [5].

The speed gain (*G*) was determined as 0.6 units per second in pilot studies to obtain desirable speed/accuracy tradeoffs. Speed magnitudes higher than *G* were clipped to *G*.

Eight target classes associated with the eight active movement classes were used in the real-time test. For each target class, six different levels of difficulty (Table 1) were employed, resulting in 48 total targets. Instead of the Shannon formulation [48] of *Index of Difficulty*, the Shannon-Welford variation [49, 50] was used which separates the influence of target distance and width to provide improved predictive power (Eq (2)). This formulation reduces to the Shannon form when *k* = 1.

$$ID={log}_{2}(\frac{W+D}{W^{k}})$$ (2)

**Table 1:** The targets distances and widths and the resulting indices of difficulty.

*Distance*	*Width*	*ID*
1	0.10	1.80
1	0.15	1.57
1	0.25	1.32
0.5	0.10	0.92
0.5	0.15	0.75
0.5	0.25	0.59

Based on the pilot studies, *k* was set to 0.5, to maximize the linearity of the relationship between the movement time and *Index of difficulty*.

The order of presentation of targets was randomized and targets were presented in four trials of 12 targets. Considering position and orientation as two Cartesian dimensions, achieving a target with width *W* required the user to maintain the cursor within a 2D Euclidean distance of W/2 of the target for a dwell time of 1s. As feedback, the cursor color changed when this distance was below *W/2*. If a target was not acquired within 15s, the task was timed out and was considered as a failure.

Each test trial was repeated using the SVM or CNN based systems in a random order, before proceeding to the next trial. Four metrics [29, 9, 33, 31]—completion rate, throughput, path efficiency, and overshoot—were computed to evaluate the control performance.

During the test, the screen was refreshed every 30ms to update the position and orientation of the cursor. The real-time computation time for the SVM based system was 3ms (2ms for the SVM and 1ms for the feature extraction), and for the CNN was 9ms. However, the same delay was used for both methods for a fair comparison. This was implemented by using the same real-time code for both methods, i.e. regardless of the method, both SVM and CNN outputs were computed in the real-time, to produce a fixed total delay.

### Statistics

The offline classification accuracies as well as the four metrics of the real-time test were compared between the CNN and SVM based schemes using one-way ANOVA tests. The significance level was set to 0.05.

## Results

### Offline results

The classification accuracies (mean ± standard error) for the CNN and SVM based systems using a 4-fold cross validation were 91.61 ± 0.39, and 90.63 ± 0.31, respectively. No significant difference (*p* = 0.059) was found between the classification accuracies of the CNN and SVM based schemes.

### Fitts’ law test results

**Fig 3:**
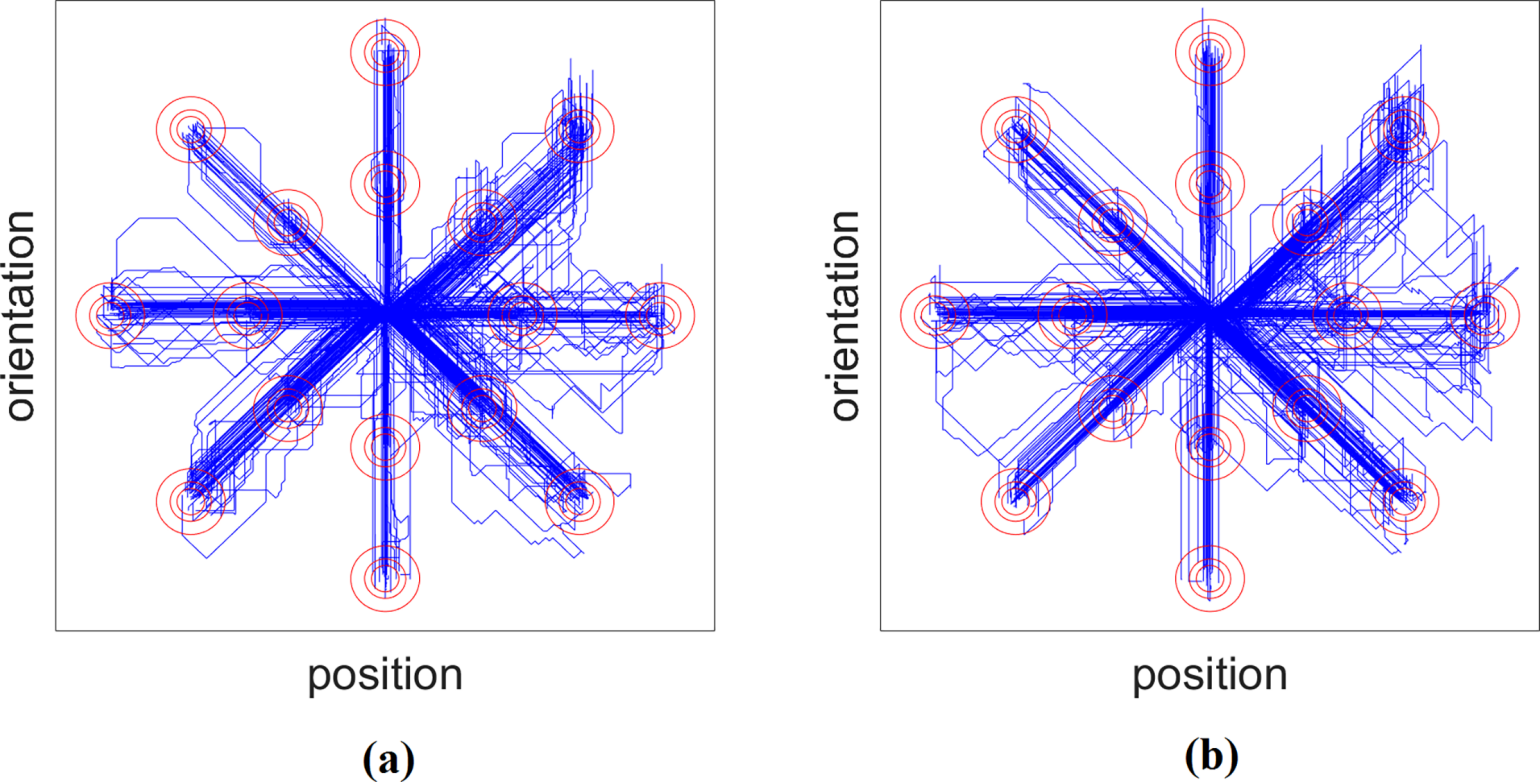
A 2D representation of the overlay of path traces for all users in the Fitts’ law test for the (a) CNN and (b) SVM based control scheme (x+: extension, x-: flexion, y+: supination, y-: pronation).

A 2D representation of the overlay of path traces for all users is plotted in Fig 3 for the CNN and SVM based control schemes.

**Fig 4:**
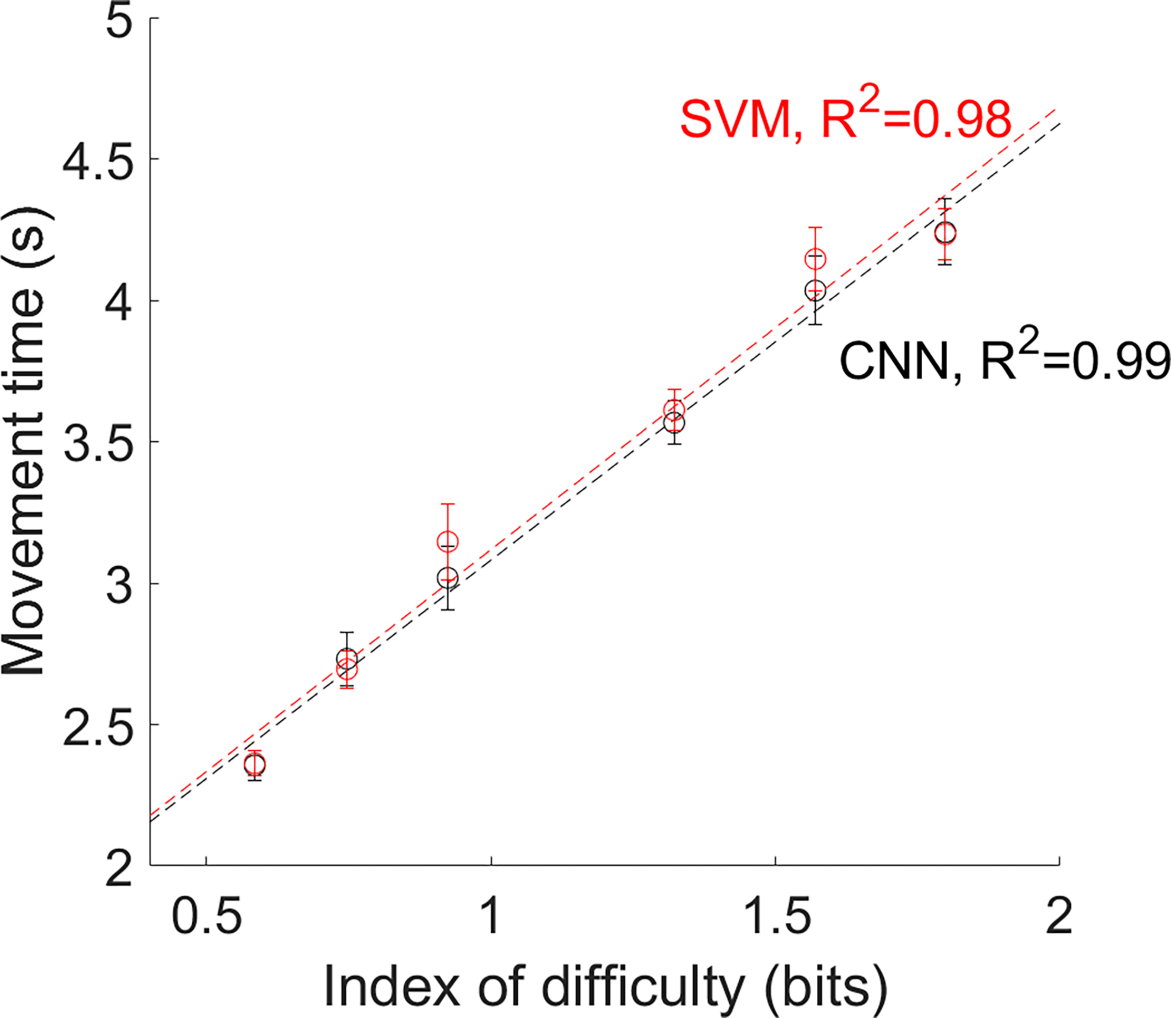
A strong linear relationship (*R*^*2*^>0.98) was found between the movement time and index of difficulty.

The average time to acquire the Fitts’ law test targets are shown in Fig 4 for the CNN and SVM based methods, along with the error bars indicating the standard error across the users. The linear regression line of best fit is also plotted for each method. The coefficient of determination (*R*^*2*^) between the average time and linear regression was higher than 0.98 for both control schemes, which indicates that the real-time test results obeyed Fitts’ law, because of the strong linear relationship between the movement time and index of difficulty.

The control performance metrics are listed in Table 2 for both systems. No significant difference (*p*>0.05) was found between the two methods in all control metrics.

**Table 2:** The Fitts’ law test metrics are presented as mean ± standard error, along with the statistical analyses results.

	*CNN*	*SVM*	*p-value*
*Throughput*	0.36 ± 0.01	0.35 ± 0.01	0.71
*Completion* *Rate (%)*	100	100	-
*Overshoot*	0.98 ± 0.01	1.00 ± 0.01	0.27
*Path* *Efficiency (%)*	91.73 ± 0.70	90.99 ± 0.72	0.46

## Discussion

This work sought to validate the viability and efficacy of CNNs for real-time myoelectric control. The results showed that although CNN was applied to the raw EMG data, it achieved offline and real-time performances no different (*p*>0.05) than those of the SVM, which has been provided with discriminating features manually extracted from the EMG data. All users were asked to report their preference regarding the control scheme, and none of them noticed any difference, so that they could not distinguish between the methods by the quality of the control. These results suggest that for motor intent estimation, CNN automated feature extraction is as effective as the combined TD and mean frequency features.

It is not clear, however if any EMG engineered feature set can outperform CNNs, as both CNN architecture design and feature engineering rely on human experts. Also, CNNs are rapidly advancing and novel configurations may improve their performance. An interesting question to answer is that if CNNs can improve the robustness of the EMG pattern recognition against changes in the EMG signal as in limb position change or electrode shift.

Based on the results, CNNs were shown to be able to automatically identify and extract EMG features that are reproducible across movements of the same class, and discriminative among different classes of movements. Because of the complexity of EMG patterns, several convolution layers are required in the network. While the first convolution layers compute low-level features, the last convolution layers extract high-level aggregated features. The higher level features are better suited for classification tasks as they combine the lower level features into a more meaningful representation of the data [51].

Both temporal and frequency content of the EMG signal are necessary for accurate motor intent estimation. This information is incorporated in the TD features (temporal as mean-absolute value, waveform length; frequency as zero-crossings, slop sign changes) and other common EMG features (autoregressive features, spectral features, entropy). The results therefore suggest that CNNs are capable of extracting complex information from EMG using several convolution layers followed by rectified linear units. The ability for CNNs and other deep learning in an automated feature extraction approach may create opportunities for studying EMG-based physiological information.

In this study, CNNs were directly applied to the raw EMG data. However, the performance may improve if the learning is performed on some EMG transformed domain coefficients, because features extracted from the new space may enhance motor intent estimation accuracy.

This work described simultaneous myoelectric control of wrist movements. During the real-time test, the subjects performed single and combined contractions, intuitively, to control the cursor. However, occasional erroneous cursor movements due to misclassification, were also observed. Most of these cases were misclassification between combined motions and their corresponding single motions (e.g. extension misclassified as combined extension-supination). This may be, in part, due to the anatomy of the forearm muscles. The muscles responsible for wrist rotation are smaller and deeper than those for wrist flexion and extension. They are also closely located and move under the skin during contractions. Therefore, rotation may cause the recorded EMG signals to be picked up by different sensors depending on the angle of rotation. This issue will be more pronounced when increasing the number of movement classes. Using intramuscular electrodes may reduce this problem by providing EMG signals from isolated muscles [52, 53].

Recently, both classification [32–34] and regression [35–40] based methods have been used to implement simultaneous control. Regression-based systems have the potential advantage of independent simultaneous control of multiple degrees of freedom (DOFs). On the other hand, classification-based methods may improve the robustness of proportional control because the output intensity is not affected by the regression noise. An obvious next step is to examine the performance of CNNs as applied to EMG regression based control.

This work was a preliminary study on the application of CNN for EMG-based motor intent estimation. Although no improvement were achieved over the classical SVM-based method, this approach is promising because CNN allows for novel configurations that may improve the performance. Furthermore, high computational complexity of CNNs can be overcome using future generations GPUs.This work validated the use of a CNN-based myoelectric system with able-bodied subjects. Although comparable relative performances have been demonstrated between algorithms for amputees [18], future evaluation of CNNs should include limb deficient subjects.

## Conclusion

A CNN-based myoelectric control scheme was proposed and tested in a real-time control task. The control performance was compared to that of a classical pattern classification system (SVM) with extraction of engineered features. The CNN based method showed no significant difference in performance from that of the SVM based approach in both offline and real-time tests results. Because the CNN was applied to the raw EMG data, these results suggest that the CNN was able to automatically derive and extract motor intent commands as effectively as the combined TD and mean frequency feature sets. Furthermore, their usability and robustness were demonstrated, for the first time, in an online Fitts’ law style tasks.

Based on these promising results, it is possible that CNNs may help to discover new motor information hidden in the EMG signal. Future work will study the impact of EMG transformations prior to CNN-based motor intent estimation, as well as its robustness to external factors, such as limb position and electrode shift. Also, given the ability of CNNs to incorporate high dimensional data, and its ability to find intrinsic features, EMG signals combined with additional data such as force myography (FMG) and accelerometer signals, may be used for improved CNN based myoelectric control.
